# Longitudinal determinants of anal intercourse among women with, and without HIV in the United States

**DOI:** 10.1186/s12905-022-01849-0

**Published:** 2022-07-14

**Authors:** Branwen Nia Owen, Rebecca F. Baggaley, Mathieu Maheu-Giroux, Jocelyn Elmes, Adaora A. Adimora, Catalina Ramirez, Andrew Edmonds, Kemi Sosanya, Tonya N. Taylor, Michael Plankey, Julie A. Cederbaum, Dominika Seidman, Kathleen M. Weber, Elizabeth T. Golub, Jessica Wells, Hector Bolivar, Deborah Konkle-Parker, Gudrun Pregartner, Marie-Claude Boily

**Affiliations:** 1 Department of Infectious Disease Epidemiology, Imperial College London, St Mary’s Campus, Norfolk Place, Praed Street, London, W2 1NY UK; 2 Department of Respiratory Sciences, University of Leicester, University Road, Leicester, UK; 3 Department of Epidemiology, Biostatistics, and Occupational Health, School of Global and Population Health, McGill University, Montreal, Canada; 4 Department of Public Health, Environments and Society, London School of Hygiene and Tropical Medicine, London, UK; 5 Department of Epidemiology, Gillings School of Global Public Health, University of North Carolina at Chapel Hill, Chapel Hill, NC USA; 6 Department of Medicine, School of Medicine, University of North Carolina at Chapel Hill, Chapel Hill, NC USA; 7 Montefiore Medical Center Bronx, New York, NY USA; 8 Department of Medicine, Division of Infectious Disease, SUNY Downstate Health Sciences University, Brooklyn, NY USA; 9 Department of Medicine, Division of Infectious Diseases, Georgetown University Medical Center, Washington, D.C., USA; 10 Suzanne Dworak-Peck School of Social Work, University of Southern California, Los Angeles, USA; 11 Obstetrics, Gynecology and Reproductive Sciences, University of California San Francisco, San Francisco, CA USA; 12 Cook County Health/Hektoen Institute of Medicine, Chicago, IL USA; 13 Department of Epidemiology, Johns Hopkins Bloomberg School of Public Health, Baltimore, MD USA; 14 Neil Hodson Woodruff School of Nursing, Emory University, Atlanta, GA USA; 15 Division of Infectious Diseases, University of Miami-Miller School of Medicine, Miami, Fl USA; 16 Department of Medicine, University of Mississippi Medical Center, Jackson, MS USA; 17 Institute for Medical Informatics, Statistics and Documentation, Medical University of Graz, Graz, Austria; 18 MRC Centre for Global Infectious Disease Analysis, Imperial College, London, UK

**Keywords:** Heterosexual, Anal sex, Women, Transmission, Prevention

## Abstract

**Background:**

Anal intercourse (AI) is not uncommon among U.S. women and, when condomless, confers a far greater likelihood of HIV transmission than condomless vaginal intercourse. We aim to identify determinants preceding AI, among women with, and women without HIV.

**Methods:**

3708 women living with (73%), and without HIV (27%) participating in the Women’s Interagency HIV Study provided sexual behavior and other data at 6-monthly visits over a median of 9 years (1994–2014). We used generalized estimating equation models to examine sociodemographic, structural and behavioral determinants reported in the visit preceding (1) AI, and (2) condomless AI.

**Results:**

AI was reported at least once over follow-up by 31% of women without, and 21% with HIV. AI was commonly condomless; reported at 76% and 51% of visits among women living without HIV, and with HIV, respectively. Women reporting AI were more likely to be younger (continuous variable, adjusted odds ratio (aOR) = 0.97, 95% confidence interval (CI):0.96–0.98), Hispanic (aOR = 1.88, CI:1.47–2.41) or White (aOR = 1.62, CI:1.15–2.30) compared to Black, and have at least high school education (aOR = 1.33, CI:1.08–1.65). AI was more likely following the reporting of either (aOR = 1.35, CI:1.10–1.62), or both (aOR = 1.77, CI:1.13–2.82) physical and sexual violence, excessive drinking (aOR = 1.27, CI:1.05–1.66) or any drug use (aOR = 1.34, CI:1.09–1.66), multiple male partners (aOR = 2.64, CI:2.23–3.11), exchange sex (aOR = 3.45, CI:2.53–4.71), one or more female sex partners (aOR = 1.32, CI:1.01–1.75), condomless vaginal intercourse (aOR = 1.80, CI:1.53–2.09), and high depressive symptoms (aOR = 1.23, CI:1.08–1.39).

**Conclusion:**

AI disproportionally follows periods of violence victimization, substance use, multiple sex partners and depression. Better prevention messaging and biomedical interventions that reduce acquisition or transmission risk are needed, but when AI occurs in the context of violence against women, as our findings indicate, focusing on gender-based violence reduction and immediate treatment to reduce HIV transmission risk is important.

**Supplementary Information:**

The online version contains supplementary material available at 10.1186/s12905-022-01849-0.

## Background

Anal intercourse (AI) is practiced by women in the U.S. with an estimated 25% of sexually active young women and girls (aged < 25 years) in North America reporting having ever practiced AI [1], and 30% of sexually active women living in 20 US cities with high HIV prevalence reported AI within the past year [2]. The likelihood of HIV transmission during condomless receptive AI may be up to 18-fold higher than during condomless receptive vaginal intercourse (VI) [3]. Given this elevated transmission risk, AI among women may substantially shape the HIV epidemic in the U.S. A mathematical modeling study suggested that four in ten new HIV infections among urban U.S. women may be attributed to condomless AI [4].

Numerous cross-sectional studies have identified correlates of AI among women in the U.S., finding that women who report AI are more likely to report multiple sexual partners [2, 5, 6], transactional sex [2, 5, 7–10], lower condom use [11–13] and sex with both men and women [2, 14]. Substance use has also frequently been found to be positively associated with AI, with AI being more common among U.S. women who report using any type of illegal drug [6, 7, 9, 11, 15, 16], or binge drinking [2, 10, 17]. Although some women do report finding AI pleasurable [18], it does seem that it may often occur under coercion or in the context of violent relationships, with women reporting AI more likely to also report rape or coerced sex [19, 20], and to have experienced intimate partner physical violence [15, 21].

While we have a fair understanding from prior studies of current behaviors and exposures associated with AI among women, cross-sectional studies are limited by the lack of clear temporality between exposures, such as violence victimization, and AI. Prospective analysis should be conducted to better understand determinants of AI and condomless AI over time among women living with or at risk for HIV infection. Using 23 years of data from the *Women’s Interagency HIV Study* (WIHS) cohort, we aimed to identify time-varying determinants of AI and condomless AI over follow-up, among both women living with and without HIV.

## Methods

### The WIHS Cohort

The WIHS was the largest, and longest prospective cohort study of HIV infection among U.S. cisgender women, comprising 3677 women living with HIV (WLHIV) and 1305 demographically similar women without HIV. Initial recruitment occurred in 1994, with further recruitment waves in 2001–02 and 2011–12 at six urban sites (Bronx, NY; Washington, DC; San Francisco, CA; Los Angeles, CA; Chicago, IL; and Brooklyn, NY). In 2013, a fourth wave expanded recruitment to sites in the Southern U.S. (Chapel Hill, NC; Atlanta, GA; Miami, FL; Birmingham, AL; and Jackson, MS [22]. Briefly, WLHIV were recruited from HIV and other clinics and the community, as were demographically-similar women without HIV. Women without HIV were eligible for inclusion if they reported recent high-risk sexual or substance use behavior, or a diagnosis of a sexually transmitted infection (STI), although eligibility criteria varied slightly for each wave (Additional file 1: Table S1). Data were collected at approximately 6-month intervals, using structured face-to-face interviews or occasionally phone interviews when necessary. Local ethical approval for collection of data on violence victimization was granted at most sites from the first round, but was first granted to the San Francisco site in 2006, and was never granted for the Los Angeles site. All other data relevant to this analysis were collected at all sites, although data on violence and depression were not collected at all visits.

All cohort participants provided written informed consent. Ethical approval for data collection was granted by review boards at each study site.

### Data analysis

We first described the proportion of visits at which AI and condomless AI were reported over follow-up. We then used bivariate and multivariable logistic regression models to examine sociodemographic, structural and behavioral determinants of our two outcomes of interest: (1) AI and (2) condomless AI. As observations were not independent, we used generalized estimating equations (GEE) to cluster standard errors at the participant level (with an exchangeable correlation structure) [23]. Visits with and without reported (1) AI and (2) condomless AI were compared using determinants measured during the prior visit. Condomless AI was defined as reporting that condoms were used ‘sometimes’ or ‘never’ during AI since the last visit, versus ‘always’.

The recall period for the outcomes of interest and the time-varying covariates was ‘since the last visit’, which was approximately six months prior if no study visits had been skipped. Our analysis was restricted to visits where the recall period of the outcome was up to approximately one year (i.e., a maximum of one skipped prior visit).

Guided by a literature-based conceptual framework, covariates of interest available in the WIHS dataset were identified and selected a priori (Additional file 1: Figure S1) [24]. Multicollinearity between covariates was assessed using the variance inflation factor [25]. Collinear variables were combined where reasonable, otherwise one was selected based on quasi information criteria (QIC) values measuring model fit [26]. If QIC varied little, the variable with fewest missing values was retained. Thus, the highly collinear variables for number of male partners and exchange sex (defined as practicing sex in exchange for money or drugs) were combined into one variable, as were sexual and physical violence victimization. Household income was chosen over the collinear housing and employment status variables based on QIC values, while crack, cocaine or heroin use was chosen over injection drug use based on number of missing values.

Time-varying covariates of interest were: HIV status (n = 23 women sero-converted during follow-up), age (continuous), marital status (married or living with a partner / not), annual household income (< $12,000/$12,000 +), physical (experienced serious physical violence) and sexual (pressured or forced to have sex) violence victimization (none/either physical or sexual/both physical and sexual), alcohol use (dichotomized at seven drinks per week, the limit above which alcohol use among women is considered to be problematic drinking per National Institute on Alcohol Abuse and Alcoholism [27]), any crack, cocaine or heroin use, the number of male partners and exchange sex (one partner and no exchange sex/multiple partners and no exchange sex/exchange sex), any female sexual partners, condomless VI (‘never’ or ‘sometimes’ using condoms during VI), and high depressive symptoms (defined as scoring 16 + on the Center for Epidemiologic Studies Depression Scale [28]).Models were additionally controlled for non-time-varying covariates measured at baseline: educational level (< high school/ high school +) and race and ethnicity (Non-Hispanic Black/Hispanic or Latina/White/Other). The small number of women who seroconverted during follow-up (n = 23) were considered as not living with HIV until documentation of seroconversion; for visits after this point their status was changed. The same covariates apart from condomless VI were used to examine determinants of condomless AI. WIHS participants with at least two follow-up visits, and for whom data on AI and each time-varying covariate were available at least once, were included in our analysis.

Ten of the 13 included covariates had missing data (all missing at < 8% of visits except for the violence covariates, missing at 39% of visits, and depressive symptoms, missing at 12%). In this context, a complete case analysis would have dropped 52% of all study visits; we therefore dealt with missing covariate values using multiple imputation. Outcome variables were not imputed. We used a joint modelling approach which uses a multivariate normal model fitted by Markov Chain Monte Carlo [29]. Along with the covariates of interest, the imputation model additionally controlled for recruitment wave and baseline study site, as missingness differed by these variables. Twenty imputed datasets were produced and combined using Rubin’s rule [30]. As a sensitivity analysis, we also performed the analysis on the subset of complete cases.

All analyses were conducted using the R statistical software [31], with “*mitml*” and “*jomo”* packages [32, 33] used for multiple imputation and the “*geepack*” package [34] used for GEE regression models.

## Results

### Study participants and visits

Of the 4982 total women recruited, 3708 women (74%) met the inclusion criteria for this analysis (Additional file 1: Figure S2). Baseline characteristics are presented in Table 1. Participants were followed for a median of 9 years (inter quartile range (IQR) = 3.0–16.5). Over a quarter (27%) of women (n = 1004) were not living with HIV at baseline; the remaining 73% of women were living with HIV at enrollment. Median baseline age was 37 years (IQR = 31–44). Over two-thirds of women described themselves as non-Hispanic Black (69%) and over a sixth as Hispanic or Latina (16%). The remainder identified as non-Hispanic White (12%) and other ethnicities and races (3%). Median income was low, with 56% having an annual household income of less than $12,000/year, and less than a third were employed (29%). Experience of violence victimization was common, with 55% reporting ever having been victims of physical, and 40% of sexual violence. The median number of lifetime male sex partners was 10 (IQR = 5–40). 43% of women reporting ever having had AI, 36% reported ever having practiced exchange sex. Most baseline characteristics did not vary by HIV status.

**Table 1:** Baseline characteristics of 3708 participants of *Women Interagency HIV Study*, stratified by HIV status at baseline

		Total N (%) or median (IQR)	WLHIV N (%) or median (IQR)	Women without HIV N (%) or median (IQR)
	TOTAL	N = 3708	N = 2704	N = 1004
Years of follow-up	Median (IQR)	9.0 (3.0–16.5)	8.0 (3.0–16.5)	13.0 (3.0–17.5)
Recruitment wave	First (1994)	1717 (46.3%)	1325 (49.0%)	392 (39.0%)
	Second (2001–02)	884 (22.8%)	547 (20.2%)	297 (29.6%)
	Third (2011–12)	328 (8.9%)	240 (8.9%)	88 (8.8%)
	Fourth (2013–15)	819 (22.1%)	592 (21.9%)	227 (22.6%)
Site	Atlanta, GA^a^	265 (7.1%)	180 (6.7%)	85 (8.5%)
	Birmingham, AL^a^	111 (3.0%)	84 (3.1%)	27 (2.7%)
	Bronx, NY	736 (19.8%)	528 (19.5%)	208 (20.7%)
	Brooklyn, NY	616 (16.6%)	458 (15.7%)	158 (15.7%)
	Chapel Hill, NC^a^	190 (5.1%)	141 (5.2%)	49 (4.9%)
	Chicago, IL	546 (14.7%)	425 (15.7%)	121 (12.1%)
	Jackson, MS^a^	111 (3.0%)	83 (3.1%)	28 (2.8%)
	Los Angeles, CA^b^	9 (0.2%)	8 (0.3%)	1 (0.1%)
	Miami, FL^a^	142 (3.8%)	104 (3.9%)	38 (3.8%)
	San Francisco, CA	419 (11.3%)	285 (10.5%)	134 (13.3%)
	Washington, DC	563 (15.2%)	408 (15.1%)	155 (15.4%)
Age in years	Median (IQR)	37 (31–44)	37 (31–44)	36 (28–43)
Race and ethnicity	Non-Hispanic Black	2562 (69.1%)	1878 (69.5%)	684 (68.1%)
	Hispanic/Latina	606 (16.3%)	436 (16.1%)	170 (16.9%)
	Non-Hispanic White	427 (11.5%)	319 (11.8%)	108 (10.8%)
	Other	113 (3.1%)	71 (2.6%)	42 (4.2%)
Sexual orientation	Heterosexual	3221 (86.9%)	2397 (88.6%)	824 (82.1%)
	Bisexual	292 (7.8%)	183 (6.8%)	109 (10.9%)
	Lesbian	151 (4.1%)	96 (3.4%)	55 (5.5%)
	*Missing*	*44 (1.2%)*	*28 (1.0%)*	*16 (1.6%)*
Education	< High school	1274 (34.4%)	948 (35.1%)	326 (32.5%)
	≥ High school	2432 (66.6%)	1755 (64.9%)	677 (67.4%)
	*Missing*	*2 (0.5%)*	*1 (0.3%)*	*1 (0.1%)*
Marital status	Married or living with partner	1269 (34.2%)	1753 (64.8%)	332 (33.1%)
	Not married or living with partner	2423 (65.3%)	937 (34.7%)	670 (66.7%)
	*Missing*	*16 (0.4%)*	*14 (0.5%)*	*2 (0.2%)*
Household annual income	< $12,000	2063 (55.6%)	1530 (56.6%)	553 (53.1%)
	≥ $12,000	1530 (41.3%)	1098 (40.6%)	432 (43.0%)
	*Missing*	*115 (3.1%)*	*76 (2.8%)*	*39 (3.9%)*
Employed	Yes	1084 (29.2%)	737 (27.3%)	347 (34.6%)
	No	2.615 (70.5%)	1961 (72.5%)	654 (65.1%)
	*Missing*	*9 (0.2%)*	*6 (0.2%)*	*3 (0.3%)*
Physical violence victimization, ever^c^	Yes	1811 (48.8%)	1302 (48.2%)	509 (50.7%)
	No	1512 (40.8%)	1123 (41.5%)	389 (38.7%)
	*Missing*	*385 (10.4%)*	*279 (10.3%)*	*106 (10.6%)*
Sexual violence victimization, ever^c^	Yes	1324 (35.7%)	970 (35.9%)	354 (35.3%)
	No	1984 (53.5%)	1444 (53.4%)	540 (53.8%)
	*Missing*	*400 (10.8%)*	*290 (10.7%)*	*110 (11.0%)*
Injection drug use, ever	Yes	881 (23.8%)	693 (25.6%)	188 (18.7%)
	No	2826 (76.2%)	2010 (74.3%)	816 (81.3%)
	*Missing*	*1 (0.0%)*	*1 (0.0%)*	*0 (0.0%)*
Number of male sex partners, ever	Median (IQR)	10 (5–40)	10 (5–40)	12 (6–35)
	*Missing*	*N* = *54*	*N* = *46*	*N* = *8*
Number of female sex partners, ever	0	2730 (73.6%)	2034 (75.2%)	696 (69.3%)
	≥ 1	962 (25.9%)	656 (24.3%)	306 (30.5%)
	*Missing*	*16 (0.4%)*	*14 (0.5%)*	*2 (0.2%)*
Anal intercourse, ever^d^	Yes	1376 (37.1%)	990 (36.6%)	386 (38.4%)
	No	1802 (48.6%)	1270 (47.0%)	532 (53.0%)
	*Missing*	*530 (14.3%)*	*444 (16.4%)*	*86 (8.6%)*
Exchange sex, ever	Yes	1337 (36.1%)	979 (36.2%)	358 (35.7%)
	No	2360 (63.6%)	1716 (63.2%)	644 (64.1%)
	*Missing*	*12 (0.2%)*	*9 (0.3%)*	*2 (0.2%)*

*IQR* interquartile range. Variables for which there is no “missing” category contain no missing values. ^a^New sites were added in the fourth recruitment wave. All other sites were added during the first recruitment wave. ^b^Most women from the Los Angeles site were excluded from this analysis as this site did not collect data on violence victimization. The 9 women included here lived in Los Angeles at baseline and subsequently moved to other sites. ^c^Violence victimization variables have many missing values, as ethical approval was not granted at the Los Angeles and San Francisco study sites. ^d^The number of missing values is high because in the first recruitment wave, women reporting no sex partners in the past 6 months were not asked whether they had ever practiced AI. In subsequent waves, all women were asked whether they had ever practiced AI

### AI over follow-up

AI was reported at least once over follow-up by nearly a quarter of women (24%) and was more commonly reported by women without HIV (31%) than by WLHIV (21%). Reporting condomless AI at least once was twice as common among women without HIV (28%) as among WLHIV (14%).

Across the whole sample, AI was reported at 5% of all visits. A male sexual partner was reported at two-thirds of visits (64%) with AI reported at 8% of those visits. At visits when a male sex partner was reported, women without HIV reported AI at 10% of visits and condomless AI at 8% of visits, compared to 7% and 4%, respectively, reported by WLHIV. AI was more often condomless among women without AI; reporting that three-quarters (76%) of AI acts were condomless while HIV-infected women reported that nearly half (51%) of AI acts were condomless. Table 2 details the proportions of visits at which time-varying covariates of interest were reported, as well as the proportions of visits at which AI was reported at the subsequent visit, among the whole sample (with proportions by HIV status reported in Additional file 1: Table S2).

**Table 2:** Time-varying covariates over follow-up, and percentages of visits at which anal intercourse and condomless anal intercourse were reported at the subsequent visit (N = 3708 over 77,257 visits)

Variable	Category	n (visits reported)/N (total visits)	% visits reported (%)	% visits AI subsequently reported (%)	% visits CAI subsequently reported (%)
Married or living with a partner	No	49,203/71,663	68.7	5.0	2.8
	Yes	22,460/71,663	31.3	5.4	3.4
*Missing = 7.2%*					
Household annual income	< $12,000	36,277/70,968	51.1	4.7	2.9
	≥ $12,000	34,691/70,968	48.9	5.5	3.1
*Missing = 8.1%*					
Violence victimization	Neither	45,225/47,245	95.7	4.4	2.5
	Either physical or sexual	1749/47,245	3.7	10.9	7.2
	Both physical and sexual	271/47,245	0.6	17.0	12.2
*Missing = 38.8%*					
Alcohol use	< 8 drinks/week	65,232/73,204	89.1	4.7	2.7
	≥ 8 drinks/week	7972/73,204	10.9	8.1	5.0
*Missing = 5.3%*					
Crack, cocaine or heroin use	No	64,887/73,267	88.6	4.6	2.7
	Yes	8380/73,267	11.4	8.8	5.5
*Missing = 5.2%*					
Number of male sex partners and exchange sex^a^	No partner, no exchange sex	25,606/71,522	35.8	0.5	0.3
	1 partner, no exchange sex	37,943/71,522	53.1	5.8	3.5
	≥ 2 partners, no exchange sex	6364/71,522	8.9	14.3	8.2
	Any exchange sex	1609/71,522	2.3	17.3	10.2
*Missing = 7.4%*					
Number of female sex partners	0	69,349/73,271	94.6	5.1	3.0
	≥ 1	3922/73,271	5.4	5.5	3.5
*Missing = 5.2%*					
Condomless VI	No	49,005/72,041	68.0	2.9	0.8
	Yes	23,036/72,041	32.0	9.7	7.5
*Missing = 6.8%*					
High depressive symptoms^b^	No	43,122/67,538	63.8	4.6	2.6
	Yes	24,416/67,538	36.2	5.9	3.5
*Missing = 12.4%*					

*AI* anal intercourse, *CAI* condomless anal intercourse, *VI* vaginal intercourse, subsequently = at next visit. ^a^Women reported only one male sex partner at 197 of 1487 visits (13.2%) where exchange sex was reported. ^b^Scores of > 15 in the Center for Epidemiologic Studies Depression Scale were defined as likely depression (36)

#### Determinants of AI, and condomless AI over follow-up

Although the unadjusted odds of AI were substantially lower among WLHIV than among women without HIV (odds ratio (OR) = 0.67; 95% confidence interval (CI): 0.54–0.83), the association did not remain after multivariable adjustment (Table 3). Adjusted odds of condomless AI, however, were lower among WLHIV (adjusted odds ratio (aOR) = 0.61; 95% CI: 0.46–0.74). AI decreased substantially with age (aOR = 0.94; 95% CI: 0.94–0.95 per one-year increase in age). After multivariable adjustment, Hispanic and White women had over 1.5 times the odds of reporting AI than Black women (e.g., aOR = 1.62; 95% CI: 1.25–2.30 among White women), while condomless AI was more likely to be reported by Hispanic women (aOR = 1.65; 95% CI: 1.25–2.20), but not by White women, relative to Black women. After multivariable adjustment, AI was slightly more likely among women who had finished high school (aOR = 1.33; 95% CI:1.08–1.65).

**Table 3:** Determinants of anal intercourse and condomless anal intercourse over follow-up among women in the *Women’s Interagency HIV Study* cohort (N = 3,708 over 69,438 visits)

		Any AI				Condomless AI			
		Univariate analysis		Multivariable analysis		Univariate analysis		Multivariable analysis	
		OR	95% CI	aOR	95% CI	OR	95% CI	aOR	95% CI
HIV status	Seronegative	Ref		Ref		Ref		Ref	
	Seropositive	**0.67**	**0.54–0.83**	1.04	0.89–1.28	**0.45**	**0.35–0.57**	**0.61**	**0.46–0.74**
*Demographic determinants*									
Age	Years, continuous	**0.94**	**0.94–0.95**	**0.97**	**0.96–0.98**	**0.94**	**0.93–0.95**	**0.96**	**0.95–0.97**
Race and ethnicity^a^	Non-Hispanic Black	Ref		Ref		Ref		Ref	
	Hispanic/Latina	**1.50**	**1.17–1.92**	**1.88**	**1.47–2.41**	**1.45**	**1.08–1.94**	**1.65**	**1.25–2.20**
	Non-Hispanic White	1.40	0.98–1.99	**1.62**	**1.15–2.30**	0.98	0.63–1.39	1.05	0.73–1.55
	Other	1.21	0.71–2.03	1.29	0.79–2.03	1.23	0.66–2.30	1.21	0.82–2.18
Education^a^	< High school	Ref		Ref		Ref		Ref	
	≥ High school	1.20	0.98–1.48	**1.33**	**1.08–1.65**	1.09	0.85–1.40	1.25	0.97–1.61
Married or living with a partner	No	Ref		Ref		Ref		Ref	
	Yes	1.12	0.94–1.33	0.88	0.76–1.06	**1.24**	**1.03–1.51**	1.10	0.93–1.32
Household annual income	< $12,000	Ref		Ref		Ref		Ref	
	≥ $12,000	**1.19**	**1.02–1.39**	1.14	0.98–1.33	1.06	0.86–1.26	0.98	0.81–1.15
Violence victimization	None	Ref		Ref		Ref		Ref	
	Either physical or sexual	**2.64**	**2.18–3.18**	**1.35**	**1.10–1.62**	**2.94**	**2.35–3.56**	**1.62**	**1.28–2.08**
	Both physical and sexual	**4.50**	**2.95–7.12**	**1.77**	**1.13–2.82**	**5.82**	**3.37–9.06**	**2.50**	**1.44–4.12**
*Behavioral determinants*									
Alcohol use	< 8 drinks/week	Ref		Ref		**Ref**		**Ref**	
	≥ 8 drinks/week	**1.91**	**1.55–2.31**	**1.27**	**1.05–1.51**	**1.96**	**1.56–2.34**	**1.31**	**1.04–1.62**
Crack, cocaine or heroin use	No	Ref		Ref		**Ref**		**Ref**	
	Yes	**2.08**	**1.69–2.52**	**1.34**	**1.09–1.66**	**2.05**	**1.61–2.62**	**1.35**	**1.02–1.78**
Number of male sex partners and exchange sex^b^	1 partner, no exchange sex	Ref		Ref		Ref		**Ref**	
	≥ 2 partners, no exchange sex	**2.64**	**2.23–3.11**	**2.10**	**1.79–2.50**	**2.49**	**2.00–3.03**	**1.91**	**1.52–2.33**
	Any exchange sex	**3.45**	**2.53–4.71**	**2.20**	**1.57–3.13**	**3.21**	**2.13–4.68**	**1.83**	**1.16–2.81**
Number of female sex partners	0	Ref		Ref		Ref		Ref	
	≥ 1	1.11	0.84–1.47	**1.32**	**1.01–1.75**	1.21	0.87–1.71	1.32	0.89–1.92
Condomless VI	No	Ref		Ref		–	–	–	–
	Yes	**3.48**	**2.95–4.02**	**1.80**	**1.53–2.09**	–	–	–	–
*Psycho-social determinants*									
High depressive symptoms^c^	No	Ref		Ref		Ref		Ref	
	Yes	**1.36**	**1.19–1.53**	**1.23**	**1.08–1.39**	**1.42**	**1.20–1.67**	**1.25**	**1.03–1.48**

Visits for which AI data were available and for which the prior visit was no longer than 12 months ago (i.e., maximum one visit skipped) were included in the analysis. Most follow-up visits (93.1%) were six months apart, with 5.3% of visits occurring after one skipped visit; applying this criterion therefore removed only 1.6% of visits. Missing values for covariates were imputed (see Methods for details). The covariates HIV status, age and race and ethnicity had no missing values. Education status was missing for 1 woman (measured at baseline). Data on marital status were missing at 4.9% of visits, household income at 5.7%, violence victimization at 39.2%, alcohol use at 3.6%, crack, cocaine, or heroin use at 3.5%, number of male partners and exchange sex at 6.0%, number of female partners at 3.5%, condomless VI at 5.2% and high depressive symptoms at 11.3% of visits

*OR* odds ratio, *aOR* adjusted odds ratio, *CI* confidence interval, *VI* vaginal intercourse. All covariates were collected over follow-up and represent since the last visit except race and ethnicity and education level, which were measured at baseline. Results in bold indicate that the 95% CI does not include the null value

^a^Variables measured at baseline. All other variables were measured over follow-up. ^b^Women reported only one male sex partner at 197 of 1487 visits (13.2%) where exchange sex was reported. ^c^Scores of > 15 in the Center for Epidemiologic Studies Depression Scale were defined as high depressive symptoms(36)

Odds of AI as well as condomless AI increased with violence victimization. Odds of AI were higher when both physical and sexual violence were reported (aOR = 2.50; 95% CI: 1.44–4.12), compared to one form of violence (aOR = 1.62; 95% CI: 1.28–2.08). Relative to non-substance users, odds of AI and condomless AI were slightly higher following periods of problematic alcohol use (AI, aOR = 1.27; 95% CI:1.05–1.51) and use of crack, cocaine, or heroin (AI, aOR = 1.34; 95% CI:1.09–1.66). Compared to women reporting one male sex partner and no exchange sex, women reporting multiple partners and no exchange sex, as well as those reporting exchange sex, had approximately two-fold adjusted odds of both AI and condomless AI. Women reporting a recent female sex partner had a slightly elevated adjusted odds of subsequent AI (aOR = 1.32; 95% CI: 1.00–1.75) but not higher adjusted odds of condomless AI, while women reporting condomless VI had nearly two-fold the odds (aOR = 1.80; 95% CI:1.53–2.09) of subsequently reporting AI. Having high depressive symptoms slightly increased the odds of both subsequent AI (aOR = 1.23; 95% CI:1.08–1.39) and condomless AI (aOR = 1.25; 95% CI:1.03–1.48).

Results were similar when the analysis was restricted to complete cases (Additional file 1: Tables S3 and S5), although 95% CIs were narrower in models using multiple imputation. Models stratified by HIV status at baseline also found similar results (Additional file 1: Tables S4, S5).

## Discussion

Using more than two decades of data from the WIHS cohort, we found that AI decreased with age and is more commonly practiced by White and Hispanic women (compared to Black women) and more educated women. We found AI to be associated with previous violence victimization, excessive drinking, drug use, likely depression, and more varied or risky sexual behaviours: multiple male partners, any female partners, transactional sex, and condomless VI. These findings concur with observations from numerous cross-sectional studies [24], but our longitudinal analysis supports the temporal order of these associations; strengthening the evidence by avoiding potential reverse causality issues.

The associations of AI which we identified here are similar to findings of other papers, including to our previous analysis which used group-based trajectory modelling to identify groups with distinct AI behaviors among WIHS participants living without HIV [35]. We found that the trajectory group reporting AI most consistently over the life course were, at baseline, more likely to identify as bisexual or lesbian, to report more male sex partners, and to report ever having experienced physical or sexual violence, compared to the group who rarely or never reported AI.

Determinants of AI and condomless AI were largely similar, although there were some interesting differences. Adjusted estimates of AI did not differ by HIV status, but condomless AI was much less commonly practiced among WLHIV. Further, while White women (compared to Black women) and women with at least a high school education were more likely to report AI, they were not more likely to practice condomless AI, implying that these women tend to use condoms more consistently. Women experiencing violence were more likely to report AI and condomless AI, but the magnitude of association with condomless AI was 1.5 times greater. This could imply that when AI occurs in the context of violence, it is more likely to be unprotected by condoms. This conjecture is supported by other studies which examined intimate partner violence directly, and found it to be associated with condomless AI [15, 19], while others have found that intimate partner violence is associated with using condoms inconsistently, regardless of sex act type [36]. Further research, ideally taking a qualitative approach, should be conducted to more thoroughly explore the relationships between violence, coercion, condom use and AI.

A main limitation of this analysis is the use of face-to-face interviews to collect data, and AI as well as condomless sex may be underreported due to social-desirability bias. Meta-analyses have found that the reporting of AI increases with confidentiality of interview method [1, 37, 38]. As reported elsewhere [6, 8], we found AI to be more common among women with higher educational attainment. We did not assess whether AI is less stigmatized among women with higher educational attainment who may therefore more accurately report AI. Second, the lack of data on violence perpetrators was a limitation, as we were unable to directly assess whether intimate partner violence predicted AI. Third, high proportions of values were missing for some indicators. The use of multiple imputation helped avoid some biases and allowed us to retain the large sample size, but also incorporated additional uncertainty in the results.

### Policy implications

As AI has been found to be fairly common among US women, clinicians should routinely include question on AI practice when assessing patients’ HIV and STI risk. In addition, in order to anal STIs, women should be offered both rectal and vaginal tests, rather than solely vaginal tests, as is currently the norm in routine STI screening [39].The determinants of AI identified in this paper can be used to improve targeting of safe sex messaging and of prevention services such as both HIV pre-exposure prophylaxis (PrEP) and post-exposure prophylaxis (PEP). However, when AI occurs in the context of violence, as these findings indicate it often might, women are unlikely to be able to insist on condom use, and may also be unable to safely access and take PrEP. As such, gender-based violence reduction interventions, may offer an additional path to reducing HIV transmission among women. These could range from individual-level approaches such as teaching teens healthy relationship skills, teaching parenting skills and engaging with families in early childhood, to structural interventions such as improving school climate and safety, and strengthening economic support for families [40].

## Supplementary Information

**Additional file 1: Table S1.** Eligibility criteria of women without HIV recruited to the WIHS cohort. **Table S2**. Percentage of visits at which time-varying covariates reported, and percentage of visits at which anal intercourse and condomless anal intercourse reported at subsequent visit. **Table S3**. Predictors of anal intercourse and condomless anal intercourse using a complete case analysis. **Table S4**. Predictors of any anal intercourse over follow-up, by HIV status, using a complete case analysis. **Figure S1**. A conceptual framework of anal intercourse among women in the U.S. **Figure S2**. Selection process of analysis sample.

## Data Availability

Access to individual-level data from the MACS/WIHS Combined Cohort Study Data (MWCCS) may be obtained upon review and approval of a MWCCS concept sheet. Links and instructions for online concept sheet submission can be found on the study website: https://statepi.jhsph.edu/mwccs/work-with-us/. Upon request, we will share the code used for this analysis with anyone with access to the relevant WIHS data.
