# Supplementary material for: Longitudinal determinants of anal intercourse among women with, and without HIV in the United States
Source: BMC Womens Health. 2022 Jul 14;22:291. doi: 10.1186/s12905-022-01849-0 (PMC9284855; doi:10.1186/s12905-022-01849-0)
Supplement: Supplementary file 1 — Additional file 1: Table S1. Eligibility criteria of women without HIV recruited to the WIHS cohort. Table S2. Percentage of visits at which time-varying covariates reported, and percentage of visits at which anal intercourse and condomless anal intercourse reported at subsequent visit. Table S3. Predictors of anal intercourse and condomless anal intercourse using a complete case analysis. Table S4. Predictors of any anal intercourse over follow-up, by HIV status, using a complete case analysis. Figure S1. A conceptual framework of anal intercourse among women in the U.S. Figure S2. Selection process of analysis sample. [file 12905_2022_1849_MOESM1_ESM.docx]

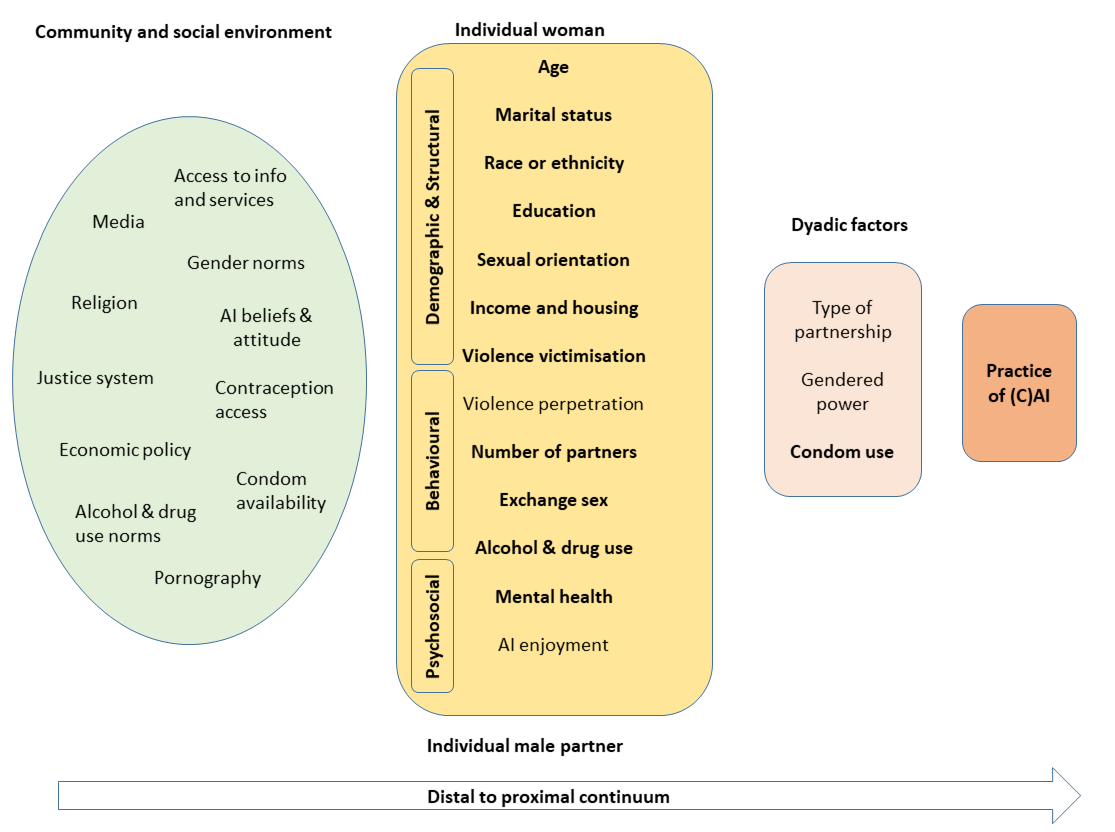


**Supplementary Figure 1.** A conceptual framework of anal intercourse practice among women in the US.

Bold font indicates covariates of interest for which data were available in the WIHS dataset. AI=anal intercourse, CAI= condomless anal intercourse, exchange sex=sex in exchange for money, drugs, goods or services.

| **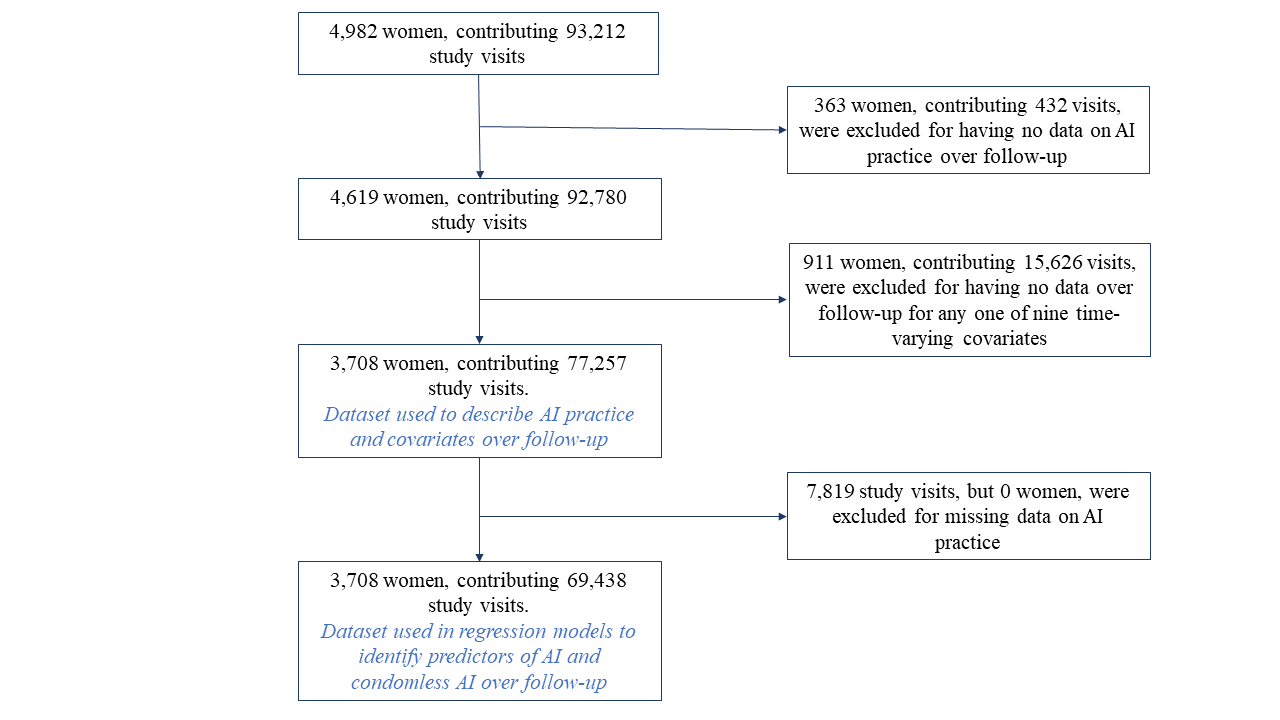** |
| --- |
| **Supplementary Figure 2.** Selection process of analysis sample |

**Supplementary Table 1.** Eligibility criteria of women without HIV recruited to the WIHS cohort.

| **Wave** | **Years recruited** | **Eligibility criteria for women without HIV** |
| --- | --- | --- |
| 1 | 1994-95 | No targeted age range. Women without HIV were recruited to match demographic characteristics of recruited women with HIV. |
| 2 | 2001-02 | No targeted age range. Reporting at least one of the following within the past year: (i) injection drug use; (ii) having a sexually transmitted disease; (iii) having unprotected sex with three or more men or protected sex with more than five men; or (iv) having exchanged sex for drugs, money, or shelter. |
| 3 | 2011-12 | Age 30-55 years and reporting at least one of the following within the past year: (i) injection drug use; (ii) having a sexually transmitted disease; (iii) having unprotected sex with three or more men or protected sex with more than five men; or (iv) having exchanged sex for drugs, money, or shelter |
| 4 | 2013-15 | Age 25-60 years and reporting at least one high-risk exposure in the preceding 5 years, e.g. STI diagnosis; sex without a condom with three or more men; sex with a condom with six or more men; trading sex; sex with an HIV-seropositive man; injection drug use or use of crack cocaine, cocaine, heroin or methamphetamine; or any partner who had any of the previously mentioned risk characteristics. |

The history of the WIHS cohort has been described in Adimora et al., 2018(9). Women with HIV were recruited from the community, and were eligible if they fell within the specified age range (in waves 3 and 4 only). Women without HIV were demographically matched to recruited women with HIV in each wave.

Women in waves 1-3 were recruited in the Bronx, NY; Washington, DC; San Francisco, CA; Los Angeles, CA; Chicago, IL; and Brooklyn, NY study sites. In 2013, a fourth wave expanded recruitment to sites in the Southern US (Chapel Hill, NC; Atlanta, GA; Miami, FL; Birmingham, AL; and Jackson, MS).**Supplementary Table 2.** Percentage of visits at which time-varying covariates reported, and percentage of visits at which anal intercourse and condomless anal intercourse reported at subsequent visit.

|  |  | Women living with HIV | | | | Women not living with HIV | | | |
| --- | --- | --- | --- | --- | --- | --- | --- | --- | --- |
| Variable | Category | n (visits reported)/N (total visits) | % visits reported | % visits AI subsequently reported | % visits CAI subsequently reported | n (visits reported)/N (total visits) | % visits reported | % visits AI subsequently reported | % visits CAI subsequently reported |
| Married or living with a partner | No | 35,845/51,556 | 69.5% | 4.3% | 2.1% | 12,887/19,442 | 66.3% | 6.9% | 4.6% |
| *Missing*=7.2% | Yes | 15,711/51,556 | 30.5% | 4.9% | 2.4% | 6,555/19,442 | 33.7% | 6.5% | 5.7% |
| Household annual income | <$12,000 | 26,834/51,061 | 52.6% | 4.3% | 2.3% | 8,976/19,247 | 46.7% | 5.9% | 4.5% |
| *Missing*=8.1% | ≥$12,000 | 24,227/51,061 | 47.4% | 4.7% | 2.1% | 10,271/19,247 | 53.4% | 7.5% | 5.5% |
| Violence victimization | Neither | 32,601/33,954 | 96.0% | 4.0% | 1.9% | 12,237/12,887 | 95.0% | 5.6% | 4.1% |
| *Missing*=38.8% | Either physical or sexual | 1,166/33,954 | 3.5% | 9.9% | 5.8% | 572/12,887 | 4.4% | 12.7% | 10.0% |
|  | Both physical and sexual | 187/33,954 | 0.5% | 14.6% | 9.6% | 78/12,887 | 0.6% | 22.2% | 18.1% |
| Alcohol use | <8 drinks/week | 47,704/52,529 | 90.8% | 4.1% | 2.0% | 16,975/20,003 | 84.9% | 6.4% | 4.7% |
| *Missing*=5.3% | ≥8 drinks/week | 4,825/52,529 | 9.2% | 8.0% | 4.1% | 3,028/20,003 | 15.1% | 8.2% | 6.4% |
| Crack, cocaine or heroin use | No | 47,175/52,579 | 89.7% | 4.1% | 2.0% | 17,260/20,014 | 86.2% | 6.0% | 4.5% |
| *Missing*=5.2% | Yes | 5,404/52,579 | 10.3% | 7.7% | 4.2% | 2,754/20,014 | 13.8% | 10.8% | 7.8% |
| Number of male sex partners  and exchange sex | No partner, no exchange sex | 19,637/51,223 | 38.3% | 0.3% | 0.1% | 5,767/19,644 | 29.4% | 0.2% | 0.1% |
|  | 1 partner, no exchange sex | 27,223/51,223 | 53.1% | 5.5% | 2.7% | 10,397/19,644 | 52.9% | 6.7% | 5.6% |
|  | ≥2 partners, no exchange sex | 3,515/51,223 | 6.9% | 14.3% | 7.3% | 2,781/19,644 | 14.2% | 14.3% | 9.1% |
| *Missing*=7.4% | Any exchange sex | 848/51,223 | 1.7% | 13.3% | 6.9% | 699/19,644 | 3.6% | 21.7% | 13.7% |
| Number of female sex partners | 0 | 50,375/52,580 | 95.8% | 4.5% | 2.2% | 18,316/20,016 | 91.5% | 6.7% | 4.9% |
| *Missing*=5.2% | ≥1 | 2,205/52,580 | 4.2% | 4.1% | 2.1% | 1,700/20,016 | 8.5% | 7.2% | 5.2% |
| Condomless VI | No | 39,350/51,661 | 76.2% | 2.8% | 0.7% | 9,262/19,720 | 47.0% | 3.2% | 1.4% |
| *Missing*=6.8% | Yes | 12,311/51,661 | 23.8% | 9.5% | 69.4% | 10,458/19,720 | 53.0% | 9.9% | 8.2% |
| High depressive symptoms^2^ | No | 30,153/48,280 | 62.5% | 3.9% | 1.8% | 12,556/18,643 | 67.3% | 6.1% | 4.5% |
| *Missing*=12.6% | Yes | 18,127/48,280 | 37.5% | 5.2% | 2.8% | 6,087/18,643 | 32.7% | 7.8% | 5.7% |

AI=anal intercourse, CAI=condomless anal intercourse, VI=vaginal intercourse, subsequently=at next visit. “Missing” refers to the percentage of visits with missing values.

**Supplementary Table 3.** Predictors of any anal intercourse and any condomless anal intercourse using a complete case analysis

|  |  | Predictors of any AI | | | | | Predictors of any condomless AI^1^ | | | |  |
| --- | --- | --- | --- | --- | --- | --- | --- | --- | --- | --- | --- |
|  |  | N women=3,531, N visits=40,370 | | | | | N women=3,531, N visits=40,371 | | | |  |
|  |  |  | Univariate analysis | | Multivariable analysis | | Univariate analysis | | Multivariable analysis | |  |
|  |  |  | OR | 95% CI | aOR | 95% CI | OR | 95% CI | aOR | 95% CI |  |
| HIV status | Seronegative |  | Ref |  | Ref |  | Ref |  | Ref |  |  |
|  | Seropositive |  | **0.67** | **0.54-0.83** | **1.05** | **0.85-1.31** | **0.45** | **0.35-0.57** | **0.61** | **0.47-0.78** |  |
| Demographic determinants |  |  |  |  |  |  |  |  |  |  |  |
| Age | Years, continuous |  | **0.94** | **0.94-0.95** | **0.97** | **0.96-0.98** | **0.94** | **0.93-0.95** | **0.96** | **0.95-0.98** |  |
| Race | Non-Hispanic Black |  | Ref |  | Ref |  | Ref |  | Ref |  |  |
|  | Hispanic/Latina |  | **1.50** | **1.17-1.92** | **1.88** | **1.47-2.41** | **1.45** | **1.08-1.94** | **1.68** | **1.25-2.26** |  |
|  | Non-Hispanic White |  | 1.40 | 0.98-1.99 | **1.61** | **1.12-2.32** | 0.94 | 0.63-1.39 | 1.05 | 0.72-1.55 |  |
|  | Other |  | 1.21 | 0.71-2.03 | 1.29 | 0.81-2.06 | 1.23 | 0.66-2.30 | 1.24 | 0.82-2.21 |  |
| Education | <High school |  | Ref |  | Ref |  | Ref |  | Ref |  |  |
|  | ≥High school |  | 1.20 | 0.98-1.48 | **1.33** | **1.08-1.64** | 1.09 | 0.85-1.40 | **1.25** | **0.97-1.61** |  |
| Married or living with a partner | No |  | Ref |  | Ref |  | Ref |  | Ref |  |  |
|  | Yes |  | 1.12 | 0.94-1.33 | 0.89 | 0.75-1.06 | **1.24** | **1.02-1.51** | 1.09 | 0.90-1.32 |  |
| Household annual income | <$12,000 |  | Ref |  | Ref |  | Ref |  | Ref |  |  |
|  | ≥$12,000 |  | **1.19** | **1.02-1.39** | 1.14 | 0.98-1.33 | 1.04 | 0.86-1.26 | 0.98 | 0.81-1.18 |  |
| Violence victimization | None |  | Ref |  | Ref |  | Ref |  | Ref |  |  |
|  | Either physical or sexual |  | **2.63** | **2.15-3.22** | **1.34** | **1.09-1.64** | **2.99** | **2.36-3.60** | **1.63** | **1.27-2.09** |  |
|  | Both physical or sexual |  | **4.55** | **2.88-7.18** | **1.77** | **1.10-2.84** | **5.85** | **3.41-10.03** | **2.48** | **1.42-4.33** |  |
| Behavioral determinants |  | |  |  |  |  |  |  |  |  |  |
| Alcohol use^2^ | <8 drinks/week |  | Ref |  | Ref |  | **Ref** |  | **Ref** |  |  |
|  | ≥8 drinks/week |  | **1.89** | **1.54-2.31** | **1.29** | **1.05-1.58** | **1.92** | **1.53-2.41** | **1.30** | **1.02-1.66** |  |
| Crack, cocaine or heroin | No |  | Ref |  | Ref |  | **Ref** |  | **Ref** |  |  |
|  | Yes |  | **2.10** | **1.69-2.59** | **1.34** | **1.07-1.69** | **2.09** | **1.63-2.68** | **1.34** | **1.02-1.78** |  |
| Number of male sex partners  and exchange sex | 1 partner, no exchange sex |  | Ref |  | Ref |  | Ref |  | **Ref** |  |  |
|  | ≥2 partners, no exchange sex |  | **2.64** | **2.23-3.13** | **2.12** | **1.78-2.53** | **2.49** | **2.02-3.07** | **1.89** | **1.50-2.39** |  |
|  | Any exchange sex |  | **3.47** | **2.53-4.75** | **2.20** | **1.54-3.15** | **3.23** | **2.16-4.81** | **1.81** | **1.15-2.85** |  |
| Number of female sex partners | 0 |  | Ref |  | Ref |  | Ref |  | Ref |  |  |
|  | 1+ |  | 1.10 | 0.83-1.47 | 1.32 | 0.97-1.78 | 1.21 | 0.85-1.72 | 1.33 | 0.91-1.92 |  |
| Condomless VI^4^ | No |  | Ref |  | Ref |  | - | - | - | - |  |
|  | Yes |  | **3.49** | **2.98-4.09** | **1.81** | **1.53-2.14** | **-** | **-** | **-** | **-** |  |
| Psycho-social determinants |  |  |  |  |  |  |  |  |  |  |  |
| High depressive symptoms^5^ | No |  | Ref |  | Ref |  | Ref |  | Ref |  |  |
|  | Yes |  | **1.36** | **1.16-1.59** | **1.22** | **1.04-1.43** | **1.42** | **1.18-1.70** | **1.27** | **1.07-1.52** |  |

95% of participants are retained in the complete case analysis compared to the multiple imputation analysis. However, only 52% of visits are retained.

OR=odds ratio, aOR=adjusted odds ratio, CI=confidence interval, VI=vaginal intercourse. All covariates were collected over follow-up and measured since the last visit except race and education level, which were measured at baseline. Results in bold indicate that the 95% CI does not include the null value.

^1^Percentage of visits when AI is reported. In total, AI was reported at 4.4% of visits among HIV-positive women and at 6.6% of visits among HIV-negative women. ^2^Dichotomized at 8 drinks/week as 8+ drink per week is considered problematic drinking among women (35). ^3^Defined as exchanging sex for money or drugs. ^4^Reporting never or sometimes versus always using condoms during VI. ^5^“High depressive symptoms” was determined by scoring the series of survey items forming the Center for Epidemiologic Studies Depression Scale. Scores of >15 were defined as high depressive symptoms(36).

##### **Supplementary Table 4:** Predictors of any anal intercourse and any condomless anal intercourse using a complete case analysis

|  |  | Women living with HIV  N=2,571, Total visits=29,126 | | | | | Women not living with HIV^1^  N=960, Total visits=11,245 | | | | |
| --- | --- | --- | --- | --- | --- | --- | --- | --- | --- | --- | --- |
|  |  |  | Univariate analysis | | Multivariable analysis | |  | Univariate analysis | | Multivariable analysis | |
|  |  |  | OR | 95% CI | aOR | 95% CI |  | OR | 95% CI | aOR | 95% CI |
| Demographic determinants |  |  |  |  |  |  |  |  |  |  |  |
| Age | Years, continuous |  | **0.94** | **0.93-0.96** | **0.97** | **0.95-0.98** |  | **0.95** | **0.94-0.96** | **0.96** | **0.95-0.98** |
| Race | Non-Hispanic Black |  | Ref |  | Ref |  |  | Ref |  | Ref |  |
|  | Hispanic/Latina |  | **1.50** | **1.09-2.06** | **1.92** | **1.42-2.61** |  | 1.48 | 0.99-2.21 | **1.83** | **1.21-2.75** |
|  | Non-Hispanic White |  | **1.39** | **0.91-2.14** | **1.70** | **1.09-2.67** |  | 1.64 | 0.88-3.04 | 1.51 | 0.84-2.71 |
|  | Other |  | 0.86 | 0.45-1.62 | 0.95 | 0.50-1.78 |  | 1.66 | 0.76-3.59 | 1.78 | 0.93-3.41 |
| Education | <High school |  | Ref |  | Ref |  |  | Ref |  | Ref |  |
|  | ≥High school |  | 1.13 | 0.88-1.45 | 1.27 | 0.99-1.63 |  | 1.33 | 0.93-1.94 | **1.47** | **1.01-2.14** |
| Married or living with a partner | No |  | Ref |  | Ref |  |  | Ref |  | Ref |  |
|  | Yes |  | **1.23** | **1.01-1.52** | 0.92 | 0.74-1.14 |  | 0.91 | 0.68-1.23 | 0.85 | 0.64-1.14 |
| Household annual income | <$12,000 |  | Ref |  | Ref |  |  | Ref |  | Ref |  |
|  | ≥12,000 |  | 1.08 | 0.89-1.30 | 1.06 | 0.88-1.28 |  | **1.35** | **1.03-1.78** | **1.29** | **0.98-1.71** |
| Violence victimization | None |  | Ref |  | Ref |  |  | Ref |  | Ref |  |
|  | Either physical or sexual |  | **2.68** | **2.08-3.46** | **1.35** | **1.06-1.73** |  | **2.44** | **1.75-3.41** | 1.30 | 0.91-1.86 |
|  | Both physical and sexual |  | **4.29** | **2.43-7.58** | **2.02** | **1.11-3.69** |  | **4.88** | **2.22-10.76** | 1.46 | 0.68-3.13 |
| Behavioral determinants |  | |  |  |  |  |  |  |  |  |  |
| Alcohol use^2^ | <8 drinks/week |  | Ref |  | Ref |  |  | Ref |  | Ref |  |
|  | ≥8 drinks/week |  | **2.18** | **1.69-2.81** | **1.50** | **1.18-1.91** |  | 1.37 | 0.97-1.93 | 1.04 | 0.74-1.46 |
| Crack, cocaine or heroin | No |  | Ref |  | Ref |  |  | Ref |  | Ref |  |
|  | Yes |  | **2.10** | **1.61-2.72** | **1.18** | **0.89-1.57** |  | **1.94** | **1.36-2.77** | **1.61** | **1.10-2.34** |
| Number of male sex partners  and exchange sex | 1 partner, no exchange sex |  | Ref |  | Ref |  |  | Ref |  | Ref |  |
|  | ≥2 partners, no exchange sex |  | **2.73** | **2.20-3.38** | **2.25** | **1.18-2.81** |  | **2.38** | **1.80-3.15** | **1.98** | **1.50-2.63** |
|  | Any exchange sex |  | **3.08** | **2.14-4.45** | **1.86** | **1.18-2.92** |  | **3.66** | **2.19-6.12** | **2.56** | **1.48-4.42** |
| Number of female sex partners | 0 |  | Ref |  | Ref |  |  | Ref |  | Ref |  |
|  | ≥1 |  | 1.02 | 0.71-1.45 | 1.38 | 0.95-2.01 |  | 1.03 | 0.66-1.61 | 1.20 | 0.77-1.89 |
| Condomless VI^4^ | No |  | Ref |  | Ref |  |  | Ref |  | Ref |  |
|  | Yes |  | **3.59** | **2.98-4.32** | **1.90** | **1.57-2.31** |  | **3.17** | **2.39-4.19** | **1.60** | **1.16-2.20** |
| Psycho-social determinants |  |  |  |  |  |  |  |  |  |  |  |
| High depressive symptoms^5^ | No |  | Ref |  | Ref |  |  | Ref |  | Ref |  |
|  | Yes |  | **1.37** | **1.12-1.67** | 1.13 | 0.93-1.36 |  | **1.44** | **1.12-1.85** | **1.42** | **1.08-1.87** |

OR=odds ratio, aOR=adjusted odds ratio, 95% CI= 95% confidence interval, Ref=referent. All variables were collected over follow-up and measured since the last visit except race and education level, which were measured at baseline. Results in bold indicate that the 95%CI does not include the null value.

Visits for which AI practice data were available and for which the prior visit was no longer than 12 months ago (i.e. maximum one visit skipped), and for which all included co-variates were available, were included in analysis. Among HIV-positive women, 86.2% of visits were retained after applying these criteria; among HIV-negative women 86.7% visits were retained.

^1^Percentage of visits when AI is reported. In total, AI was reported at 4.4% of visits among HIV-positive women and at 6.6% of visits among HIV-negative women. ^2^Dichotomized at 8 drinks/week as 8+ drink per week is considered problematic drinking among women (35). ^3^Defined as exchanging sex for money or drugs. ^4^Reporting never or sometimes versus always using condoms during VI. ^5^“High depressive symptoms” was determined by scoring the series of survey items forming the Center for Epidemiologic Studies Depression Scale. Scores of >15 were defined as high depressive symptoms(36).

##### **Supplementary Table 5**: Predictors of condomless AI practice over follow-up, by HIV status using a complete case analysis

|  |  | HIV-seropositive  N=2,571, Total visits=29,126 | | | | | HIV-seronegative  N=960, Total visits=11,244 | | | | |
| --- | --- | --- | --- | --- | --- | --- | --- | --- | --- | --- | --- |
|  |  |  | Univariate analysis | | Multivariable analysis | |  | Univariate analysis | | Multivariable analysis | |
|  |  |  | OR | 95% CI | aOR | 95% CI |  | OR | 95% CI | aOR | 95% CI |
| Demographic determinants |  |  |  |  |  |  |  |  |  |  |  |
| Age | Years, continuous |  | **0.94** | **0.93-0.96** | **0.97** | **0.95-0.99** |  | **0.95** | **0.93-0.96** | **0.96** | **0.95-0.98** |
| Race | Non-Hispanic Black |  | Ref |  | Ref |  |  | Ref |  | Ref |  |
|  | Hispanic/Latina |  | **1.33** | **0.90-1.97** | **1.74** | **1.19-2.54** |  | **1.58** | **1.02-2.46** | **1.86** | **1.18-2.92** |
|  | Non-Hispanic White |  | **0.76** | **0.47-1.24** | 0.96 | 0.59-1.56 |  | 1.60 | 0.85-3.02 | 1.47 | 0.80-2.72 |
|  | Other |  | 0.61 | 0.25-1.47 | 0.68 | 0.30-1.56 |  | 1.83 | 0.84-3.02 | 1.75 | 0.86-3.27 |
| Education | <High school |  | Ref |  | Ref |  |  | Ref |  | Ref |  |
|  | ≥High school |  | 1.01 | 0.74-1.38 | 1.26 | 0.92-1.72 |  | 1.16 | 0.77-1.74 | 1.38 | 0.92-2.08 |
| Married or living with a partner | No |  | Ref |  | Ref |  |  | Ref |  | Ref |  |
|  | Yes |  | 1.24 | 0.96-1.59 | 0.92 | 0.72-1.18 |  | 1.18 | 0.86-1.61 | 1.02 | 0.76-1.38 |
| Household annual income | <$12,000 |  | Ref |  | Ref |  |  | Ref |  | Ref |  |
|  | ≥$12,000 |  | 0.84 | 0.65-1.08 | 0.86 | 0.67-1.11 |  | 1.24 | 0.93-1.65 | 1.12 | 0.85-1.47 |
| Violence victimization | None |  | Ref |  | Ref |  |  | Ref |  | Ref |  |
|  | Either physical or sexual |  | **3.15** | **2.33-4.26** | **1.39** | **1.02-1.91** |  | **2.61** | **1.79-3.81** | 1.44 | 0.96-2.16 |
|  | Both physical and sexual |  | **5.88** | **2.91-11.85** | **2.53** | **1.26-5.07** |  | **5.67** | **2.38-13.46** | 1.90 | 0.85-4.25 |
| Behavioral determinants |  | |  |  |  |  |  |  |  |  |  |
| Alcohol use^2^ | <8 drinks/week |  | Ref |  | Ref |  |  | Ref |  | Ref |  |
|  | ≥8 drinks/week |  | **2.17** | **1.60-2.94** | 1.30 | 0.95-1.77 |  | 1.38 | 0.98-1.94 | 1.11 | 0.78-1.59 |
| Crack, cocaine or heroin | No |  | Ref |  | Ref |  |  | Ref |  | Ref |  |
|  | Yes |  | **2.12** | **1.55-2.90** | 1.05 | 0.72-1.55 |  | **1.78** | **1.21-2.63** | **1.57** | **1.06-2.31** |
| Number of male sex partners  and exchange sex | 1 partner, no exchange sex |  | Ref |  | Ref |  |  | Ref |  | Ref |  |
|  | ≥2 partners, no exchange sex |  | **2.87** | **2.16-3.83** | **2.15** | **1.58-2.94** |  | **1.76** | **1.29-2.41** | **1.47** | **1.09-1.99** |
|  | Any exchange sex |  | **3.20** | **1.85-5.51** | **1.87** | **0.93-3.77** |  | **2.60** | **1.46-4.64** | **1.81** | **1.00-3.26** |
| Number of female sex partners | 0 |  | Ref |  | Ref |  |  | Ref |  | Ref |  |
|  | ≥1 |  | 0.95 | 0.61-1.47 | 1.11 | 0.68-1.82 |  | **1.76** | **1.29-2.41** | 1.37 | 0.82-2.31 |
| Condomless VI^4^ | No |  | Ref |  | Ref |  |  | Ref |  | Ref |  |
|  | Yes |  | **10.23** | **8.30-12.60** | **5.97** | **4.76-7.49** |  | **2.60** | **1.46-4.64** | **3.47** | **2.45-4.91** |
| Psycho-social determinants |  |  |  |  |  |  |  |  |  |  |  |
| High depressive symptomes^5^ | No |  | Ref |  | Ref |  |  | Ref |  | Ref |  |
|  | Yes |  | **1.54** | **1.21-1.95** | 1.21 | 0.96-5.07 |  | **1.43** | **1.09-1.89** | **1.39** | **1.07-1.80** |

See Supplementary Table 4 for table footnotes
